# NOVEL BEHAVIORAL INTERVENTIONS TARGETING COGNITIVE-MOTOR NEUROPLASTICITY IN OLDER ADULTS

**DOI:** 10.1093/geroni/igac059.1550

**Published:** 2022-12-20

**Authors:** Tanvi Bhatt, Susan Hughes, Susan Hughes

## Abstract

Comparatively little is known about the ability of physical activity to improve or maintain cognitive function in older adults. Our Midwest Roybal Center uses social/behavioral theory to promote engagement in physical activity in order to improve this outcome. In 2019, we received funding to establish a Multimodal Connectomics Core that enabled investigators to examine concurrent changes in cognitive function and brain connectivity by providing access to fMRI imaging. The Connectomics Core enabled investigators for the first time to examine cognitive function and changes in brain connectivity simultaneously by providing access to fMRI imaging as well as MRI data storage and data analysis. It enabled measuring of standardized participant outcomes selected from the NIH toolbox. This Symposium describes findings from the first three completed pilots and discusses their implications for the field. The first pilot by Bronas examined effect of a 6-month home-based walking program in people chronic kidney disease on cognitive function and intervention induced neuroplasticity compared to usual care. The second pilot by Marquez adapted the Fit & Strong! program to test the combined impact of physical activity and interactive health education for managing mood on cognitive function among older adults with depression. The third pilot by Bhatt examined the effectiveness of 4 weeks of exergaming combined explicit cognitive training on behavioral measures of fall-risk (physical and cognitive) and their associations with brain integrity measures. The discussant will initiate discussion based on outcomes of these pilots and future directions for developing them into larger studies or clinical translation.

